# In-office dental bleaching in adolescents using 6% hydrogen peroxide with different application tips: randomized clinical trial ^*^

**DOI:** 10.1590/1678-7757-2023-0216

**Published:** 2023-10-27

**Authors:** Taynara de Souza CARNEIRO, Michael Willian FAVORETO, Michel Wendlinger Cantanhede FERREIRA, Laís Giacomini BERNARDI, Heloísa Forville de ANDRADE, Matheus Coelho BANDECA, Alessandra REIS, Laura CEBALLOS GARCÍA, Alessandro Dourado LOGUERCIO

**Affiliations:** 1 Universidade Estadual de Ponta Grossa Departamento de Dentística Restauradora Ponta Grossa Brasil Universidade Estadual de Ponta Grossa , Departamento de Dentística Restauradora , Ponta Grossa , Brasil .; 2 Universidad Rey Juan Carlos Facultad de Ciencias de la Salud Madrid España Universidad Rey Juan Carlos , Facultad de Ciencias de la Salud , IDIBO, Madrid , España .

**Keywords:** Tooth Bleaching, Hydrogen Peroxide, Adolescent, Clinical Trial, Bleaching Agents

## Abstract

Despite the availability of in-office bleaching gels with a 6% concentration of hydrogen peroxide (HP), these gels have not been evaluated in younger patients. They are commercially available with a tip, associated or not with a brush, where the tip with a brush spreads the gel over the entire surface to have a smaller thickness (thin layer) since the manufacturers indicate the application of a thin layer of gel.

**Objective:**

This randomized, split-mouth, double-blind clinical trial evaluated the efficacy of in-office bleaching with 6% HP in adolescents using different application tips, as well tooth sensitivity (TS) and aesthetic self-perception.

**Methodology:**

Sixty participants were randomized for 6% HP self-mixing bleaching gel tip design: without brush and with brush. In-office bleaching was performed in 3 sessions of 50 minutes. Color change was evaluated using a digital spectrophotometer (ΔE _ab_ , ΔE _00_ , and ΔWI _D_ ) and color guide (ΔSGU), the absolute risk and intensity of TS with a visual analogue scale and aesthetic self-perception with the oral aesthetic scale (a=0.05).

**Results:**

The groups achieved similar bleaching regardless of the application tip (p>0.05). However, only for ΔWI _D_ , a significant mean difference (MD) was observed in the third week (MD 2.3; 95% CI 1.2 to 3.3; p < 0.001) and at one month (MD 1.6; 95% CI 0.6 to 2.6; p < 0.03) favoring the tip without brush. Regarding TS, 45% in the tip-without-brush group and 33% in the tip-with-brush group reported TS (odds ratio 0.61; 95% CI 0.29 to 1.28; p<0.02), with low TS intensity (MD 0.05; 95% CI -0.06 to 0.17; p>0.36). All patients reported improved aesthetic self-perception after bleaching (MD -1.3; 95% -1.8 to -0.9; p<0.001).

**Conclusions:**

Regardless of the tip used bleaching with 6% HP achieved a bleaching efficacy and improved the aesthetic self-perception. However, a lower risk of TS for application using the tip with brush was observed.

## Introduction

At-home bleaching, a technique for vital teeth, consists of applying a bleaching gel in low concentrations, distributed in individual trays and performed by the patient. ^1 , 2^ However, while some adolescents find the trays very easy to use, others consider this procedure a little difficult, especially when excess gel needs to be removed. ^3^ In-office bleaching is an option that does not depend on the patient’s cooperation and provides a faster result. ^4 , 5^ However, since in-office bleaching requires higher concentrations of bleaching gel [up to 40% hydrogen peroxide (HP)], higher levels of tooth sensitivity (TS) are reported. ^6^

TS is explained by the inflammatory response of the pulp, which is a consequence of HP penetrating the tooth structure and reaching the pulp chamber. ^7 , 8 , 9 , 10^ TS after bleaching may be expressed as a momentary sharp pain or discomfort. It is important to highlight that post-bleaching TS is transient and usually disappears as the inflammatory response subsides. ^7 - 10^ However, in certain cases, TS can lead patients to discontinue treatment. ^7^

This deleterious effect is expected to be even more relevant in younger patients (children and adolescents), as their teeth are more permeable to external stimuli due to maturation. ^11 , 12^ These patients also have a larger pulp chamber compared with older patients, ^13^ creating the potential for more intense TS. Moreover, they express a greater preference for lighter teeth than older patients. ^14^ Aesthetic procedures can improve the patient’s emotional well-being and self-esteem ^15 , 16^ and, among these procedures, tooth bleaching is the least invasive technique, ^17^ improving color. ^18 , 19^ However, more than 68% of the members of an important academy of pediatric dentistry reported that they do not provide vital bleaching for young patients. ^20^ Currently, the most suitable technique for young patients is the use of low-concentration at-home bleaching. ^21^

Several manufacturers have introduced low-concentration (HP 6–20%) in-office bleaching gels to the market, ^22 - 27^ and only a few studies have been performed in younger patients, usually evaluating in-office bleaching gel at a concentration of around 20–25% HP. ^28 , 29^ Interestingly, despite the availability of in-office bleaching gels with a concentration lower than 20% (i.e., 6%), these gels have not been evaluated in younger patients. ^22 , 30 , 31^ These in-office bleaching gels are marketed with a tip, with or without brush. The conventional tip (without brush) is the most commonly used, however, among its variations, there is one with an applicator brush, which spreads the gel over the entire surface to obtain a smaller thickness (thin layer) and increases contact with the surface, since manufacturers recommend applying a thin layer of gel. ^32^ It has been established that the thinner the layer used, ^33^ the lower the amount of bleaching gel used, ^8 , 9^ the lower the diffusion, ^34^ and the lower the degree of TS. ^32^

A recent *in vitro* study ^8^ tested the effectiveness of various application tips for 6% HP. Results showed that when the tip with a brush was used, the gel penetrated less into the pulp chamber and required approximately three times less gel compared with the tip without brush. However, when evaluating color change, a small difference was detected, favoring the tip without brush. Although *in vitro* studies are necessary to create hypotheses, they are not sufficient to make clinical recommendations. Therefore, to the best of the authors’ knowledge, no clinical studies have been conducted in adolescents to evaluate the bleaching effect of using different application tips with 6% HP bleaching gel in an in-office technique.

Therefore, this study aimed to evaluate the efficacy of in-office bleaching with 6% HP in adolescents using different application tips, as well as the risk and intensity of TS and aesthetic self-perception. We tested the following four null hypotheses: when performing in-office bleaching with 6% HP in adolescents, the use of different application tips 1) will not affect color change; 2) will not affect the absolute risk of TS; 3) will not affect the intensity of TS; and 4) will not affect aesthetic self-perception.

## Methodology

### Study design

The study was a randomized, split-mouth, double-blind clinical trial with a 1:1 allocation ratio. The description of the experimental design is in accordance with the specifications of the Consolidated Standards of Reporting Trials (CONSORT). ^35^ This study was approved by the Research Ethics Committee of the State University of Ponta Grossa, PR, Brazil (4,825,578) and registered in the Brazilian Clinical Trials Registry (RBR-274vf96).

### Recruitment

All participants were recruited via social media. The authors designed attractive visual content for dissemination on Instagram ^**®**^ , respecting specific eligibility criteria. Along with these posts, a link was shared to a form with fields for providing names and contact details. Once participants had completed the form, they were added to a list. The authors then contacted them to schedule the initial assessment and verify whether participants met the established inclusion and exclusion criteria. This approach consisted of sharing posts on the Instagram ^**®**^ feed and stories of the research group’s user account (@bleachingbond). The authors and other members of the research team also reposted this content to further amplify its reach.

### Eligibility criteria

Inclusion criteria: Adolescents aged 12 to 16 years, with vital teeth, no periodontal disease, carious lesions, endodontic treatment, in good oral and general health, and with both upper canines colored A2 or darker, according to the VITA Classical Shade Guide (VITA Zahnfabrik, Bad Säckingen, Germany). Moreover, their guardians read and signed an informed consent form before their inclusion in the study.

Exclusion criteria: Participants who had undergone previous tooth bleaching, had previous TS, were on chronic medication, used fixed orthodontic appliance or prosthesis, had gingival recession, parafunction, discoloration due to tetracycline or fluorosis, were pregnant or lactating women, smoked, or had visible cracks in their teeth.

All data were collected from September 2021 to January 2022 at the State Univesity of Ponta Grossa.

### Study intervention

**Figure 1:**
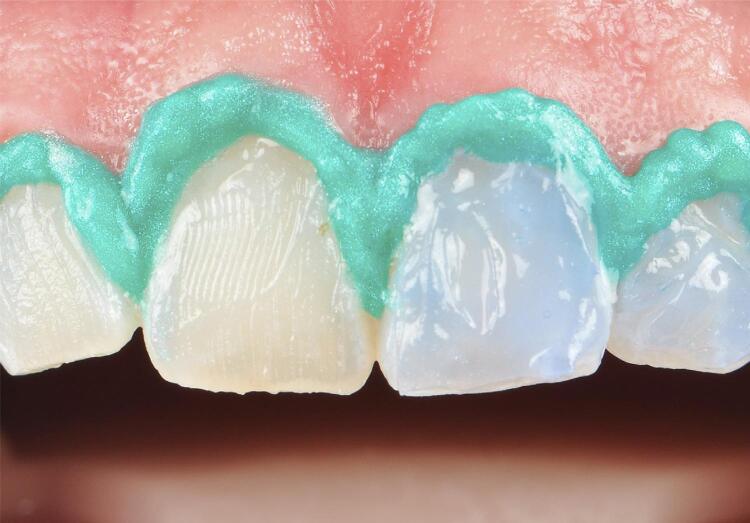
Demonstration of the amount of gel applied to the tooth surface. Patient’s right hemiarch, tip with a brush and patient’s left hemiarch, tip without brush (varying according to randomization)

Two weeks before bleaching, participants underwent prophylaxis to remove extrinsic stains. The ArcFlex retractor (FGM, Joinville, SC, Brazil) was placed. The gingiva was protected by applying the Top Dam light-curing resin (FGM, Joinville, SC, Brazil). The operator then opened the envelope defining the hemiarch and started bleaching, using a tip with or without brush (Sulzer Mixpac™, Sulzer Ltd., Winterthur, Switzerland). The bleaching gel Whiteness HP Automixx 6% (FGM, Joinville, SC, Brazil) was applied ( Figure 1 ). The treatment consisted of three sessions with an interval of seven days between them and each session lasted 50 minutes, according to the manufacturer’s recommendations.

### Outcomes

#### Color

Two operators calibrated performed color evaluation in the study, according to ISO/TR 28642, ^36^ with at least 85% agreement for the kappa statistic. Color was recorded initially, weekly for three weeks and one month after the end of treatment.

To evaluate the subjective color, the VITA Classical (VITA Zahnfabrik, Bad Säckingen, Germany) and VITA Bleachedguide 3D-MASTER (VITA Zahnfabrik, Bad Säckingen, Germany) scales were used, and the color change was evaluated by the variation of the VITA scale units (ΔSGU). ^15 , 29 , 30 - 32^ The objective evaluation was performed using the VITA Easyshade digital spectrophotometer (VITA Zahnfabrik, Bad Säckingen, Germany), ^15 , 22 , 30 , 31^ which was always calibrated before each evaluation, according to the CIELAB system. In order to standardize the evaluation with the spectrophotometer, a guide was made with condensation silicone (Perfil, Coltene, Rio de Janeiro, RJ, Brazil) on the anterior maxillary teeth, and the middle third of the right and left canines was perforated with a circular scalpel (Biopsy Punch, Miltex, York, NJ, USA) with the diameter corresponding to the active tip of the spectrophotometer. The color change recorded before and during all periods evaluated was estimated using the following formulas: $\Delta E_{ab}=[(\Delta {{L^{*})}^{2}+{\left(\Delta a^{*}\right)}^{2}+{\left(\Delta b^{*}\right)}^{2}]}^{1/2}$ , ^37^ and $\Delta E_{00}=[(\Delta L/kLSL{)}^{2}+(\Delta C/{kCSC{)}^{2}+(\Delta H/kHSH{)}^{2}+RT\left(\Delta C^{*}\Delta H/{SC}^{*}SH\right)]}^{1/2}$ ; ^38^ and the Whiteness Index for Dentistry was estimated by $WI_{D}=0.551\times L-2.324\times a-1.1\times b$ . Moreover, the changes in WI _D_ caused by each step were estimated by subtracting the values observed at each evaluation time from the values of the previous step (ΔWI _D_ ). ^18^

## Tooth sensitivity

To assess the absolute risk and intensity of TS, participants were instructed to record their sensitivity using a visual analogue scale (VAS; 0–10) ^30 - 32^ at each tooth bleaching session. Regarding risk, any value greater than zero represented the presence of TS, expressed as percentage, and intensity was measured in cm (worst-case scenario). Zero corresponded to no TS and ten corresponded to severe TS. The highest result was always recorded with a vertical line immediately after, up to one hour after, up to 24 hours after, and up to 48 hours after the bleaching session. The right and left hemiarches were always evaluated separately.

## Aesthetic self-perception

Aesthetic self-perception was assessed using the orofacial esthetic scale (OES), ^39^ which contains eight items. Participants were instructed to respond by marking with an "X" how satisfied they were with each of the eight aesthetic aspects, using a numerical scale (0–10), where zero represented very dissatisfied and ten represented very satisfied. The scale was delivered to be answered before the start of bleaching and after the end of all treatments.

## Sample size

The primary outcome of this study was to assess the efficacy of color change (∆E _ab_ ). Considering a standard deviation of 4.25 for 6% HP, ^15^ with an equivalence limit of 2.70, ^40^ a study power of 90%, and alpha of 5%, a minimum of 54 volunteers per group was required ( *https://www.sealedenvelope.com* ). A sample of 60 participants was used, totaling 120 hemiarches.

## Randomization, allocation concealment, and implementation

Using a split-mouth design, in this study, a simple randomization was performed via the website www.sealedenvelope.com to determine the sequence of application of the respective experimental groups. According to this randomization process, each specified group was enclosed in an opaque, sequentially numbered envelope. Consequently, each envelope had instructions detailing the order in which the experimental groups should be applied to a hemiarch. Given the split-mouth design, the second experimental group was consistently assigned to the second hemiarch.

Throughout the study, the initial randomized group started the process with the right upper hemiarch, while simultaneously the left upper hemiarch underwent bleaching together with the second group. The allocation sequence was disclosed immediately before the bleaching procedure in the first session and this same allocation sequence was followed in the subsequent sessions (implementation). This procedure was conducted by a researcher who was not directly involved in any of the experimental phases.

## Blinding

As this was a double-blind study, neither the evaluator nor the participant knew how the bleaching procedure was performed (tip with or without brush). Due to the different application methods evaluated during the bleaching procedure, the operator could not be blinded.

## Statistical analysis

The statistician was blinded for both groups and the analysis involved all participants and followed the intent-to-treat protocol. Two one-sided t-tests for paired samples (TOST-P) were performed to test the equivalence of the study groups at the different evaluation points. Regarding the evaluation of color change, a paired Student’s t-test was used for all scales and evaluation time points. The absolute risk of TS was compared using the McNemar test. Odds ratios were also estimated, as well as the confidence interval (CI) and Spearman correlation. The intensity of TS was analyzed using the paired Student’s t-test, and differences between the groups were detected by the Pearson correlation. Aesthetic self-perception was assessed using the paired Student’s t-test. All statistical tests were performed with an alpha of 5%.

## Results

### Characteristics of the included participants

**Figure 2:**
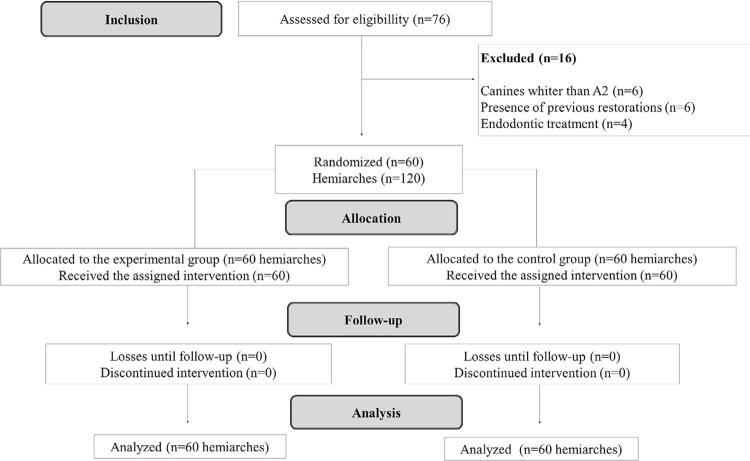
CONSORT flowchart of the study design phases, including allocation and inclusion criteria

According to the inclusion criteria, of the 76 volunteers evaluated, only 60 were included in the study. During the bleaching treatment, there was no loss of participants ( Figure 2 ). Table 1 presents their characteristics.

**Table 1:** Baseline characteristics of the participants included in this clinical trial

Groups (number of patients)	Tip without brush (n=60)		Tip with a brush (n=60)
Baseline color (Classical SGU), median (minimum value:maximum value)*	10 (7:11)		11 (7:13)
Sex (women), %		40 (67%)	
Mean age (women/men), years old		14.6 ± 1.39	

*Abbreviations: SGU: shade guide unit measured by the VITA Classical scale.

### Color

One month after the bleaching procedure, no significant differences were observed between the two groups in terms of the values obtained in the subjective scales (VITA Classical: MD 0.0; 95% CI -0.3 to 0.3; p > 0.91; and VITA Bleachedguide: MD −0.1; 95% CI −0.4 to 0.2; p > 0.49; Table 2 ) or the objective scales (ΔE _ab_: MD −0.3; 95% CI −1.4 to 0.7; p > 0.48; and ΔE _00_: MD −0.3; 95% CI −1.1 to 0.4; p > 0.38; Table 3 ). This shows the equivalence of bleaching efficacy between the groups. In the results determined by ΔWI _D_ , no statistical difference was observed between the groups after the first (MD 1.6; 95% CI -0.1 to 0.3; p=0.07) and second weeks (MD 1.9; 95% CI -0.05 to 3.8; p>0.07; Table 3 ). However, a significant difference was observed between the groups at the third week (MD 2.3; 95% CI 1.2 to 3.3; p<0.001) and after one month (MD 1.6; 95% CI 0.6 to 2.6 *;* p<0.03; Table 3 ), favoring the tip without brush.

**Table 2:** Means and standard deviations of the subjective assessment of color change and the mean difference (95% CI)

Color evaluation tool		Groups		Mean difference (95% CI)	Equivalence	p-value**
	Evaluated time	Tip without brush	Tip with brush		[p-value]*	
Vita Classical (ΔSGU)	Baseline vs. 1 week	0.6 ± 1.3	0.8 ± 1.5	-0.2 (-0.5 to 0.1)	Yes; p < 0.01	0.15
	Baseline vs. 2 weeks	2.1 ± 1.9	2.2 ± 2.1	-0.0 (-0.5 to 0.4)	Yes; p < 0.01	0.81
	Baseline vs. 3 weeks	3.6 ± 2.1	3.6 ± 2.4	-0.1 (-0.4 to 0.3)	Yes; p < 0.01	0.71
	Baseline vs. 1 month	3.7 ± 2.2	3.7 ± 2.3	0.0 (-0.3 to 0.3)	Yes; p < 0.01	0.91
Vita Bleachedguide (ΔSGU)	Baseline vs. 1 week	0.7 ± 1.3	0.8 ± 1.5	-0.2 (-0.4 to 0.04)	Yes; p < 0.01	0.10
	Baseline vs. 2 weeks	2.3 ± 2.0	2.6 ± 2.5	-0.3 (-0.6 to 0.04)	Yes; p < 0.01	0.09
	Baseline vs. 3 weeks	3.8 ± 2.2	3.9 ± 2.4	-0.1 (-0.5 to 0.2)	Yes; p < 0.01	0.51
	Baseline vs. 1 month	3.8 ± 2.2	3.9 ± 2.3	-0.1 (-0.4 to 0.2)	Yes; p < 0.01	0.49

*The p-value reported is the larger of the two p-values from the upper and lower one-sided tests (TOST-P test);**Paired t-test.

**Table 3:** Means and standard deviations of the objective assessment of color change and the mean difference (95% CI)

Color evaluation tool		Groups		Mean difference (95% CI)	Equivalence	p-value**
	Evaluated time	Tip without brush	Tip with a brush		[p-value]*	
CIELAB (ΔE _ab_ )	Baseline vs. 1 week	6.0 ± 3.4	6.4 ± 4.4	−0.4 (−1.4 to 0.7)	Yes; p<0.01	0.50
	Baseline vs. 2 weeks	7.3 ± 3.2	7.3 ± 3.4	0.1 (−1.0 to 1.2)	Yes; p<0.01	0.88
	Baseline vs. 3 weeks	8.6 ± 3.2	8.5 ± 3.6	0.2 (−0.8 to 1.1)	Yes; p<0.01	0.73
	Baseline vs. 1 month	8.2 ± 2.8	8.5 ± 3.8	−0.3 (−1.4 to 0.7)	Yes; p<0.01	0.48
CIEDE (ΔE _00_ )	Baseline vs. 1 week	4.2 ± 2.6	4.5 ± 3.3	−0.3 (−1.1 to 0.4)	Yes; p<0.01	0.41
	Baseline vs. 2 weeks	4.9 ± 2.5	5.0 ± 2.7	−0.1 (−0.8 to 0.6)	Yes; p<0.01	0.74
	Baseline vs. 3 weeks	5.7 ± 2.4	5.6 ± 2.7	0.1 (−0.6 to 0.8)	Yes; p<0.01	0.81
	Baseline vs. 1 month	5.5 ± 2.2	5.8 ± 2.9	−0.3 (−1.1 to 0.4)	Yes; p<0.01	0.38
Whiteness index (ΔWI _D_ )	Baseline vs. 1 week	5.5 ± 4.9	3.9 ± 5.9	1.6 (−0.1 to 0.3)	Yes; p<0.01	0.07
	Baseline vs. 2 weeks	8.9 ± 6.8	7.0 ± 6.6	1.9 (−0.05 to 3.8)	Yes; p<0.01	0.06
	Baseline vs. 3 weeks	9.5 ± 5.4	7.2 ± 5.8	2.3 (1.2 to 3.3)	Yes; p<0.01	0.00004
	Baseline vs. 1 month	10.5 ± 6.3	8.9 ± 6.4	1.6 (0.6 to 2.6)	Yes; p<0.01	0.03

*The p-value shown is the higher of the two p-values from the upper and lower one-sided tests (TOST-P test); **Paired t-test.

### Tooth sensitivity

TS was transient up to 24 hours and was not recorded at 48 hours. The absolute risk of participants presenting TS was 45% for the with brush group and 33.3% for the without brush group ( Table 4 ). For TS, the odds ratio was 0.61 (95% CI 0.29 to 1.28) and, consequently, a statistical difference favoring the with brush group was detected (p < 0.02; Table 4 ). The Spearman correlation coefficient was moderate and significant for binary data pairs (p < 0.0001; r=0.78). Regarding the intensity of TS, the average for the worst-case scenario was less than one unit on a 10-unit scale for both groups. The mean difference between the groups was 0.05 (95% CI −0.06 to 0.17); therefore, there was no statistical difference (p > 0.36). The correlation coefficient was moderate and significant (p < 0.0001; 0.90).

**Table 4:** Matched tabulation of the absolute risk of tooth sensitivity for both groups, along with the odds ratio and 95% CI

		Tip without brush			Odds ratio (95% CI)
		Positive	Negative	Total	
Tip with a brush	Positive	20	0	20	0.61 (0.29 to 1.28)
	Negative	7	33	40	
	Total	27	33	60	

McNemar test (p=0.023); Spearman correlation between paired data (r=0.78; p<0.0001).

### Aesthetic self-perception

No significant difference was observed when comparing the two groups (p > 0.05). However, for all eight items evaluated, there was a statistical difference after the bleaching procedure (p < 0.001; Table 5 ), with the greatest mean difference with 95% CI for the item “Color of your teeth”: −3.4 (−4.0 to −2.9) ( Table 5 ).

**Table 5:** Means and standard deviations of the orofacial aesthetic scale

Variable	Mean±SD		Mean difference (95% CI)	p-value*
	Before	After		
Facial appearance	7.7 ± 2.4	9.0 ± 2.1	−1.3 (−1.8 to −0.7)	<0.001
Facial profile appearance	6.9 ± 2.3	8.4 ± 1.7	−1.5 (−2.0 to −1.0)	<0.001
Lips appearance, smile, visible teeth	6.9 ± 2.2	8.7 ± 1.7	−1.8 (−2.4 to −1.1)	<0.001
Appearance of the tooth row	7.5 ± 2.3	8.8 ± 1.6	−1.2 (−1.8 to −0.7)	<0.001
Tooth shape	7.6 ± 2.4	8.9 ± 1.7	−1.3 (−1.9 to −0.7)	<0.001
Tooth color	5.3 ± 2.2	8.7 ± 1.7	−3.4 (−4.0 to −2.9)	<0.001
Appearance of the gums	8.2 ± 1.8	8.9 ± 1.5	−0.8 (−1.2 to −0.4)	<0.001
General feeling about the face, mouth, and teeth	7.5 ± 1.9	8.8 ± 1.9	−1.3 (−1.8 to −0.9)	<0.001

*Paired t-test.

## Discussion

The results of this study showed that 6% HP, despite the difference between the tips used, promoted significant bleaching, with a lower TS pattern and a significant difference in aesthetic self-perception after the procedure among the adolescents evaluated. This finding is of significant importance, especially since, as explained in the Introduction section, a higher prevalence of TS is expected in younger patients due to the greater permeability of their teeth. ^11 , 12^

The 6% HP applied in the study uses self-mixing tips that combine the two phases of the bleaching gel with different application tips and can be found in two ways. Considering these facts, regarding color change, this study used different evaluation parameters to better validate the results. Two subjective scales were used, the VITA Classical, ^15 , 29 , 30 , 32^ which has been used in clinical studies for a long time, allowing us to make more comparisons, and the VITA Bleachedguide 3D-MASTER, which is the proper scale for bleaching, with lighter color guides. ^15 , 31^ For the objective evaluation, the VITA Easyshade spectrophotometer was used, which also allowed us to compare with previous studies. ^15 , 22 , 30 , 31^

Significant bleaching was detected for both study groups compared with baseline values. For the subjective (VITA Classical and VITA Bleachedguide) and objective (∆E _ab_ and ∆E _00_ ) scales, there was no statistical difference between the groups. The bleaching variations observed in this study are in line with other studies that used 6% HP for bleaching and measured color change subjectively ^15 , 30 , 31^ or objectively. ^15 , 30 , 31^ However, it is worth mentioning that when the ∆WI _D_ (Whiteness Index for Dentistry *)* formula was used, a more significant bleaching was observed after three weeks and one month, favoring the without brush group. The ∆WI _D_ is a more recent formula recommended to assess the level of whiteness after tooth bleaching, with a lower probability of error than the previous one. ^18^

The favorable bleaching results for the without brush group can be attributed to the amount of gel used. Figure 1 shows a noticeable difference in gel volume favoring the without brush group. This outcome is in line with expectations, since a previous *in vitro* study evaluating various application tips for 6% HP gel showed that the tip without brush significantly enhanced the bleaching effect. ^8^ This was largely due to the fact that it required three times as much gel as the tip with a brush, as previously observed. ^8 , 9^ In fact, the outcomes of this study are in line with the results of a recent clinical investigation that showed a more pronounced bleaching effect when a larger volume of bleaching gel was applied to the tooth surface, as opposed to a smaller volume of gel. ^33^ However, the absolute value of ∆WI _D_ should be analyzed carefully ^18 , 19^ and the perceptibility and acceptability thresholds of ∆WI _D_ should be verified to establish whether the difference is detected by the observer and whether it is clinically relevant.

When assessing the 50:50% perceptibility threshold for the ∆WI _D_ , the values observed in this study had an average difference of twice 0.72, a value that is perceptible to the average calibrated observer. ^18 , 19^ However, when the 50:50% acceptability threshold was assessed, this difference was not clinically important. In other words, the value obtained in this study was lower than 2.62, which means that this difference is lower than the acceptable value for a patient. ^18 , 19^ This leads the author of this study to accept the first null hypothesis, since, although some significant differences were observed between the groups (∆WI _D_ ), they were below the acceptability threshold for bleaching procedures. ^18 , 19^

One of the most common adverse effects observed during and after tooth bleaching is TS, and many alternatives have been studied to reduce this undesirable outcome, especially in adolescents, as aforementioned in the Introduction section. Since TS occurs due to the ability of HP to penetrate the tooth structure and cause an inflammatory response in the pulp, ^7 , 8 , 9^ it is expected that the higher the concentration of HP, the greater the TS. ^23 - 27^ This expectation was indirectly confirmed by the lower absolute risk of TS observed in this study. On average, 38% of the study participants reported TS, compared with the 83% to 90% in previous studies that applied higher concentrations of HP. ^23 , 24 , 27^ However, the without brush group had a significantly higher absolute risk of TS, and these results led the authors to reject the second null hypothesis. These results are in line with an *in vitro* study ^8^ in which the application of 6% HP with a brush resulted in lower penetration of the bleaching gel compared with the without brush group, mainly because a smaller amount of gel was dispensed into the former. According to Fick’s second law, the diffusion of HP is smaller when a small volume of bleaching gel is applied. ^34^ This was recently showed in a clinical study ^33^ that found a lower risk of TS when using a smaller volume of bleaching gel compared with a larger one.

Comparing the results of this study with the literature, some differences in terms of the absolute risk of TS can be observed. ^31^ For example, according to a study, ^31^ only 6.3% of participants reported TS when 6% HP was applied in in-office bleaching. These differences can be attributed to variations in the commercial brands evaluated, the technical application, and other aspects. However, from the authors’ point of view, the most relevant factor is the age of participants. While this study evaluated young participants, the other study ^31^ included adult patients. Since younger participants have a larger pulp chamber ^13^ and more permeable teeth, ^11 , 12^ a low concentration is a good option for this age group. It is worth mentioning that the TS intensity recorded in this study was generally lower (below 1) in both groups, in line with the previous study, ^31^ which led the authors to accept the third null hypothesis.

One of the most important results of this study is the assessment of aesthetic self-perception, since it is a patient-reported outcome that characterizes specific influences of oral health on patients’ lives and this assessment is already considered essential. ^16^ Although we found no significant differences between the groups in all parameters evaluated, participants showed great satisfaction after tooth bleaching, especially when they were asked about the color of their teeth. This led the authors to reject the fourth null hypothesis and is a strong indicator that tooth bleaching, even at low concentrations, should be considered a good option for young patients. Considering that young patients are concerned about the social aspect of their appearance; ^17^ tooth bleaching can improve their emotional well-being and self-esteem. ^17^

Despite the favorable results in this study regarding in-office bleaching with 6% HP, it should be noted that the same level of bleaching efficacy should not be expected, especially when 6% HP is applied using a tip with a brush compared with more concentrated in-office bleaching gels, as previously tested in both *in vitro* ^8^ and in a clinical setting. ^15 , 30^ Therefore, we can hypothesize that conducting an additional session may improve the bleaching results, similar to what is achieved in high-concentration in-office bleaching. However, even with teeth that are not so white, participants still achieved aesthetic satisfaction, as shown by the results of the questionnaire. This outcome is significant, ^16^ especially considering the characteristics of the target population (children and adolescents).

Low-concentration in-office bleaching provided promising results. The use of 6% HP for the in-office technique has some advantages over at-home bleaching. In-office bleaching does not depend on the patient’s collaboration and is easier to be performed, as it does not require individualized trays, which adolescents find difficult to use. ^3^ Moreover, in-office bleaching is completed in only three 50-minute sessions of 50 minutes, whereas at-home bleaching with 6% HP usually requires daily application for 14 to 21 days, for one hour and 30 minutes. ^9^ The color change results in this study, regardless of the tip used, suggest that 6% HP is a good option for in-office bleaching, especially when used with a tip with a brush, which reduces the amount of gel used, ^8^ thus reducing treatment costs.

Some limitations need to be described. Our study evaluated only one commercial brand of bleaching gel, and future studies should evaluate different HP 6% bleaching gels available in clinical settings. Another limitation is the bleaching protocol, which made it difficult to compare this study with others, given the variability in application time or number of sessions, the association with light, and other factors, ^15 , 22 , 25 , 30 , 31^ requiring further studies to assess whether these factors could influence the results of this study.

## Conclusion

The use of different tips (with or without brush) for the application of 6% hydrogen peroxide in in-office bleaching in adolescents resulted in a good bleaching, regardless of the tip. A lower risk of tooth sensitivity was observed for the tip with a brush and bleaching with 6% hydrogen peroxide improved patients’ aesthetic self-perception.
